# Obituary

**Published:** 1914-06-13

**Authors:** 


### Obituary.

The committee of the London Hospital regret the death of one of the hospital's honoured physicians, Dr. Herman. Dr. Herman was appointed assistant obstetric physician in 1876, and although he retired from active work, becoming consulting physician in 1903, he has always had the same keen interest in the work of the hospital as he had when he was in active service there. Dr. Herman was one of the best known authorities in the Empire on all diseases connected with women.

